# Autoimmune Thyroid Disease and Female Fertility: Does Anti-TPO Accelerate Ovarian Aging?

**DOI:** 10.3390/jcm14228024

**Published:** 2025-11-12

**Authors:** Sefa Arlıer, Sadık Kükrer

**Affiliations:** Department of Obstetrics and Gynecology, Adana City Hospital, University of Health Science, 01230 Adana, Turkey

**Keywords:** Anti-TPO, ovarian reserve, AMH, AFC, infertility, autoimmune thyroid disease, PCOS, BMI

## Abstract

**Background/Objectives**: Thyroid autoimmunity, particularly anti-thyroid peroxidase antibodies (anti-TPO), has been implicated in reduced fertility and diminished ovarian reserve. However, the stratified effects of anti-TPO across age groups, body mass index (BMI) categories, and polycystic ovary syndrome (PCOS) status remain unclear. This study aims to investigate the association between anti-TPO positivity and ovarian reserve markers—antral follicle count (AFC), anti-Müllerian hormone (AMH), and follicle-stimulating hormone (FSH)—in euthyroid infertile women. **Methods**: This retrospective study included 1460 infertile women aged 18–45 years, evaluated between 2022 and 2025. Participants were categorized based on anti-TPO levels (≥9 vs. <9 IU/mL) using Beckman Coulter-DXI 800 analyzer, which uses chemiluminescent immunoassays to measure results. BMI (<30 vs. ≥30 kg/m^2^), and PCOS status. Age was categorized into five strata (18–25, 25–30, 30–35, 35–40, and 40–55 years), and <35 vs. ≥35 years. Linear regression models were used to assess the impact of anti-TPO on AMH and AFC within each subgroup. Additional logistic regression was performed to evaluate the odds of diminished ovarian reserve (DOR: AMH < 1 ng/mL or AFC < 5) after adjusting for age, BMI, and TSH. **Results**: Anti-TPO positivity (17.6% prevalence) was significantly associated with reduced AMH (1.47 ± 1.52 vs. 3.33 ± 3.03 ng/mL, *p* < 0.0001), reduced AFC (8.18 ± 5.06 vs. 15.88 ± 8.18, *p* < 0.0001), and elevated FSH (9.40 ± 6.21 vs. 8.06 ± 4.79 mIU/mL, *p* = 0.001). These associations remained significant in non-obese and PCOS-negative subgroups. Regression models revealed stronger associations in younger women (<35 years) and showed significant Anti-TPO × Age and Anti-TPO × BMI interactions. Logistic regression confirmed Anti-TPO ≥ 9 IU/mL as a strong predictor of diminished ovarian reserve (AMH < 1 ng/mL: OR = 3.13; AFC < 5: OR = 6.48). ROC analysis indicated modest predictive ability (AUC: 0.665–0.694), and path modeling confirmed direct effects of Anti-TPO on AMH and AFC independent of TSH or BMI. **Conclusions:** Elevated Anti-TPO levels are independently associated with diminished ovarian reserve in euthyroid women, particularly in younger, non-obese, and PCOS-negative individuals. Anti-TPO may serve as a useful biomarker in fertility risk assessment and personalized reproductive counseling, even in the absence of overt thyroid dysfunction.

## 1. Introduction

The ovary is a complex endocrine organ, and its function is closely influenced by systemic immune and hormonal homeostasis. Recent studies suggest that anti-ovarian autoantibodies, cross-reactivity of thyroid antibodies with ovarian tissue, and shared autoimmune pathways may impair folliculogenesis and lead to diminished ovarian reserve (DOR) [1,2,3]. DOR is commonly evaluated using serum FSH, AMH levels, and AFC, all of which are reliable predictors of reproductive lifespan and responses to assisted reproductive technologies. Several authors have reported lower AMH levels and AFC values in anti-TPO-positive women, suggesting a potential pathogenic role for thyroid autoimmunity (TAİ) in ovarian aging [4,5,6].

Autoimmune thyroiditis, most commonly marked by elevated anti-thyroid peroxidase (Anti-TPO) antibodies, is one of the most prevalent autoimmune conditions among women of reproductive age [7,8,9].

Anti-TPO is frequently detected in euthyroid women undergoing infertility workup and has been implicated in adverse reproductive outcomes including miscarriage, subfertility, and poor in vitro fertilization (IVF) response [8,10,11,12]. However, the underlying mechanism remains poorly understood, and its impact on quantitative ovarian reserve parameters such as AMH, AFC, and FSH remains under investigation.

Emerging evidence suggests that thyroid autoimmunity may impair folliculogenesis through immune-mediated mechanisms, local inflammation, or direct interaction with ovarian tissue [13,14,15]. Moreover, its effect may be modified by clinical conditions such as PCOS and obesity, which independently influence hormonal milieu and follicular dynamics [13,14]. Yet, the interplay between Anti-TPO, BMI, PCOS status, and age in shaping ovarian reserve has not been comprehensively explored. Therefore, a large-scale, multivariate evaluation of Anti-TPO’s impact on AMH, AFC, and FSH across age, BMI, and PCOS subgroups is critically needed to guide early fertility assessment and targeted clinical interventions.

This study aims to fill this knowledge gap by evaluating the association between Anti-TPO antibody levels and ovarian reserve markers across distinct clinical subgroups, while systematically accounting for the effects of BMI and PCOS. By disentangling these overlapping factors, we aim to clarify the role of thyroid autoimmunity in female reproductive aging and identify vulnerable subpopulations for early intervention.

## 2. Materials and Methods

### 2.1. Study Design and Setting

This retrospective cross-sectional study was conducted at the Reproductive Endocrinology and Infertility Unit of the University of Health Sciences, Adana City Hospital, Turkey, between January 2022 and February 2025. Ethical approval was obtained from the institutional review board (IRB No: 10.04.2025-442), and informed consent was secured from all participants. The study adhered to the Declaration of Helsinki.

### 2.2. Study Population and Eligibility Criteria

A total of 1460 women aged between 18 and 45 years who applied to the Reproductive Endocrinology and Infertility Unit for evaluation and treatment were included. All participants underwent a detailed infertility workup, including hormonal evaluation, ovarian reserve assessment, and TVUS imaging.

### 2.3. Inclusion Criteria

The study participants were women aged 18 to 45 diagnosed with primary or secondary infertility. Eligibility was restricted to patients with verified euthyroid state, according to institutional reference limits for (TSH; 0.4–4.5 mIU/L) and (FT4; 0.9–1.7 ng/dL). Inclusion required extensive clinical and laboratory data, encompassing measurements of Anti-TPO, AMH, AFC, FSH, LH, TSH, E2, and BMI. Additionally, PCOS diagnosis was based on the Rotterdam criteria (2003, ESHRE/ASRM). Thyroid and reproductive hormone reference ranges were determined according to the Endocrine Society Clinical Practice Guidelines (2016).

Patients were excluded if diagnosed with overt hypothyroidism or hyperthyroidism, had a documented history of systemic autoimmune diseases, or had previously undergone thyroid-specific pharmacological treatment. Further exclusion criteria encompassed a history of ovarian surgery, endometriosis, chromosomal anomalies, malignancies, or endocrine problems not associated with PCOS. Cases with incomplete hormonal or ultrasound records were excluded to maintain the dataset’s validity and reliability.

### 2.4. Data Collection

Data were extracted from the hospital’s electronic health system and cross-validated by two independent researchers. Variables included demographics (age, BMI, infertility duration). The term ‘reproductive parameters’ refers specifically to AMH, AFC, FSH, LH, E2, and PRL levels, which were assessed as key indicators of ovarian function, and ultrasonographic (AFC) findings.

#### Subgroup Definitions

Participants were stratified by anti-TPO levels (≥9 vs. <9 IU/mL; cutoff reference based on the anti-TPO kits used: 9 IU/mL) and BMI (obese: ≥30 kg/m^2^; non-obese: <30 kg/m^2^), and PCOS status (PCOS+, PCOS−). Serum total testosterone was measured using a chemiluminescent immunoassay (Beckman Coulter DxI 800) with an analytical sensitivity of 0.09 ng/mL, intra-assay CV 4.5%, and inter-assay CV 6.2%. SHBG was determined by immunoassay and the Free Androgen Index (FAI) calculated as 100× total testosterone/SHBG. Biochemical hyperandrogenism was defined as total testosterone above the assay-specific upper limit and/or FAI > 5 [6]. Anti-TPO levels were measured using a Beckman Coulter-DXI 800 analyzer, which employs chemiluminescent immunoassay. To isolate immunologic effects, BMI and PCOS were selectively excluded from specific subgroup analyses.

Hormonal assays were performed during the early follicular phase (days 2–5 of the cycle). Anti-TPO was measured using ELISA; AMH via electrochemiluminescence; FSH, LH, TSH, and E2 via automated chemiluminescent assays. AFC was determined by high-resolution transvaginal ultrasound (Mindray DC-80, 7.5 MHz probe) by a single expert sonographer. Follicles measuring 2–10 mm were bilaterally counted.

Primary outcomes were continuous AMH and AFC values. Diminished ovarian reserve (DOR) was defined as AMH < 1 ng/mL or AFC < 5. Anti-TPO ≥ 9 IU/mL defined antibody positivity. Participants were stratified by Anti-TPO status, age (<35 vs. ≥35 years), BMI (<30 vs. ≥30 kg/m^2^), and PCOS diagnosis.

### 2.5. Statistical Analyses

Analyses were conducted using SPSS version 28 (IBM Corp., Armonk, NY, USA) and Python (pandas, matplotlib, statsmodels) https://www.python.org/, accessed on 5 November 2025. A *p*-value < 0.05 was considered statistically significant. Continuous variables were tested for normality using the Shapiro–Wilk test and visual inspection of Q–Q plots. Variables showing non-normal distribution (Age, AFC, AMH, FSH, LH, E2, TSH) were expressed as the median (IQR) and analyzed using the Mann–Whitney U test. Normally distributed parameters (BMI, FT3, FT4) were expressed as the mean ± SD and analyzed by Student’s *t*-test. All regression coefficients, odds ratios, and correlation coefficients were reported with 95% confidence intervals (95% CI).

Spearman correlations evaluated Anti-TPO associations with AMH, AFC, FSH, and E2. Linear regression models predicted AMH and AFC using Anti-TPO, adjusting for age, BMI, and TSH. Interaction terms (Anti-TPO × Age, × BMI, × PCOS) assessed effect modification.

Logistic regression models predicted DOR (Model A: AMH < 1, Model B: AFC < 5) including Anti-TPO, age, BMI, and TSH. ROC curves assessed Anti-TPO predictive performance for AMH < 1 ng/mL, AFC < 5, and FSH > 10 mIU/mL. Cut-offs were based on established endocrinology guidelines.

ROC analysis for anti-TPO predicting diminished ovarian reserve (AMH < 1 ng/mL) yielded AUC 0.68 (95% CI 0.64–0.72, *p* < 0.001), indicating a moderate but statistically significant discriminatory ability. The optimal cutoff point determined by the Youden index was 9.3 IU/mL, corresponding to sensitivity 68% and specificity 62%.

Interaction Analysis: Interaction terms (Anti-TPO × Age, Anti-TPO × BMI, Anti-TPO × PCOS) were included to evaluate modification of Anti-TPO effects.

### 2.6. Sample Size and Power

Power calculations using G*Power 3.1 indicated that for detecting small-to-moderate effect sizes (f^2^ = 0.02), a sample of 395 subjects is sufficient for 80% power at α = 0.05. Our final sample size (*n* = 1460) surpassed this threshold.

Data Quality Assurance: All data entries were cross-validated by two researchers. Variables with >5% missingness were excluded. Sensitivity analyses confirmed robustness of results when excluding incomplete records.

### 2.7. Study Limitations

This study has several important limitations that warrant consideration. First, the retrospective design restricts the ability to infer causality between Anti-TPO positivity and diminished ovarian reserve. While significant associations were observed, longitudinal data are necessary to establish temporal or causal relationships.

Second, AMH and AFC measurements were cross-sectional and not repeated over time. As such, we could not assess the trajectory of ovarian reserve decline or determine whether Anti-TPO positivity accelerates the rate of decline longitudinally.

Third, potential unmeasured confounders—including other autoimmune markers (e.g., anti-thyroglobulin, anti-ovarian antibodies), vitamin D levels, and inflammatory cytokines—were not evaluated. These factors may influence both thyroid autoimmunity and ovarian reserve and should be considered in future research.

Fourth, inter-cycle variability in AFC, a known limitation in ultrasound-based follicle assessment, could not be fully controlled despite timing measurements during the early follicular phase (Day 2–5).

Finally, while the Anti-TPO cutoff of ≥9 IU/mL provided statistical clarity, Cut-off: 9 IU/mL (own laboratory kits reference value), it may not reflect standard thresholds used across different laboratories or populations. Variability in assay sensitivity and reference ranges may affect the external validity and generalizability of these findings. Future prospective, multi-center studies incorporating dynamic hormonal assessments and broader immunologic profiling are needed to confirm and expand upon these results.

### 2.8. Results

In this study cohort of 1460 infertile women, 17.6% (*n* = 257) were classified as Anti-TPO positive (≥9 IU/mL), and 82.4% (*n* = 1203) were Anti-TPO negative (<9 IU/mL). Comparative analysis between groups revealed several statistically significant differences in reproductive and endocrine parameters. The mean age was slightly higher in the Anti-TPO positive group (32.60 ± 5.75 years) than in the Anti-TPO negative group (31.44 ± 5.66 years), although this difference did not reach statistical significance (*p* = 0.554). Although the mean age was slightly higher in the anti-TPO-positive group, this difference, while not statistically significant, could still have minor clinical relevance due to the nonlinear relationship between age and ovarian reserve decline. Adjusted regression analyses confirmed that anti-TPO positivity remained significantly associated with lower AMH and AFC after controlling for age, indicating an independent autoimmune contribution. Similarly, BMI values were comparable between groups (26.37 ± 3.68 vs. 26.15 ± 3.47 kg/m^2^, *p* = 0.564), and no significant difference was found in the proportion of obese women (BMI ≥30 kg/m^2^; 14.8% vs. 17.7%, *p* = 0.213). The prevalence of polycystic ovary disease (PCOD) was significantly lower in the Anti-TPO positive group (8.5%) compared to the Anti-TPO negative group (26.6%) (*p* = 0.001), indicating a possible inverse relationship between thyroid autoimmunity and the PCOS phenotype. In terms of ovarian reserve markers, both antral follicle count (AFC) and anti-Müllerian hormone (AMH) levels were significantly reduced in Anti-TPO positive women. AFC was nearly halved (8.18 ± 5.06 vs. 15.88 ± 8.18; *p* < 0.001), and AMH levels were markedly lower (1.47 ± 1.52 vs. 3.33 ± 3.03 ng/mL; *p* < 0.001). These results strongly suggest an association between thyroid autoimmunity and diminished ovarian reserve (DOR), even in euthyroid individuals.

Conversely, FSH levels were significantly elevated in Anti-TPO positive women (9.40 ± 6.21 vs. 8.06 ± 4.79 mIU/mL; *p* < 0.001), consistent with a compensatory response to reduced ovarian reserve. Interestingly, LH levels were significantly lower in the Anti-TPO positive group (5.92 ± 3.84 vs. 6.64 ± 4.41 mIU/mL; *p* = 0.015), while estradiol (E2) levels did not significantly differ (*p* = 0.735).

Regarding thyroid function parameters, TSH levels were significantly higher in the anti-TPO-positive group (2.21 ± 1.11 vs. 1.92 ± 0.82 mIU/mL; *p* = 0.001), although all participants remained within euthyroid limits. No significant differences were observed in free T3 (*p* = 0.283) or free T4 (*p* = 0.072) levels.

Our analysis indicated a negative correlation between anti-TPO and AMH, with a correlation coefficient of −0.38 (*p* < 0.001). Additionally, anti-TPO was negatively correlated with AFC, with a negative correlation of −0.36 (*p* < 0.001). In contrast, anti-TPO showed a positive correlation with FSH of +0.11 (*p* = 0.028). No significant correlation was observed (Table 1).

**Figure 1:**
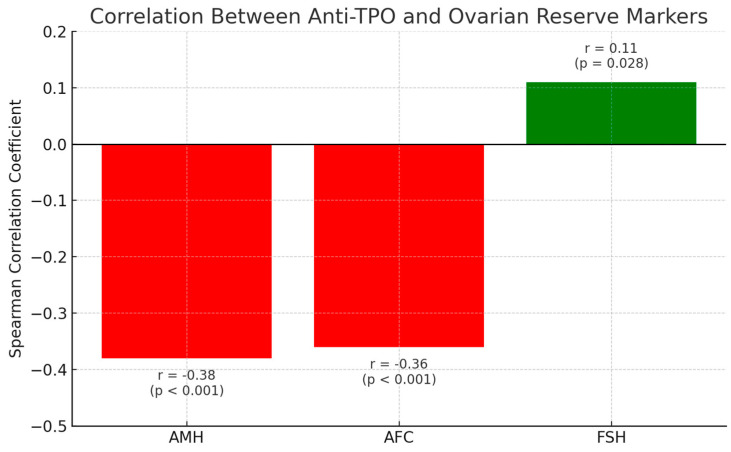
Correlation between anti-TPO and ovarian reserve markers.

Multivariate linear regression showed a strong negative relationship between anti-TPO levels and both AMH and AFC, regardless of age and BMI. In non-obese women (BMI < 30), higher anti-TPO levels were linked to lower AMH (β = −0.003, *p* < 0.001) and AFC (β = −0.014, p < 0.001). In non-obese women (BMI < 30), anti-TPO was negatively associated with AMH (β = −0.003, *p* < 0.001) and AFC (β = −0.014, *p* < 0.001). In obese women (BMI ≥ 30), the association remained significant but weaker (AMH, β = −0.005, *p* = 0.001; AFC, β = −0.023, *p* < 0.001). When interaction effects were examined, the combination of anti-TPO and age had a significant influence on AMH levels (*p* < 0.001) and AFC levels (*p* < 0.001). The interaction between anti-TPO and BMI was also significant for AMH (*p* < 0.001). These findings suggest that the impact of thyroid autoimmunity on ovarian reserve is age- and BMI-dependent, with more pronounced effects in younger, non-obese women. Anti-TPO positivity was more frequent in women aged ≥ 35 (*p* = 0.003) and among those with lower AFC values (*p* < 0.001) (Figure 1).

Spearman correlation coefficients illustrating the relationships between anti-TPO antibody levels and ovarian reserve markers: anti-Müllerian Hormone (AMH), antral follicle count (AFC), and and follicle-stimulating hormone (FSH). Negative correlations were observed between anti-TPO and both AMH (r = −0.38, *p* < 0.001) and AFC (r = −0.36, *p* < 0.001), indicating potential autoimmune-mediated impairment of ovarian reserve. A weak positive correlation was found between anti-TPO and FSH (r = 0.11, *p* = 0.028). These patterns were more pronounced in younger (<35 years) and non-obese patients, supporting the hypothesis that thyroid autoimmunity may be an early marker of reproductive compromise.

**Figure 2:**
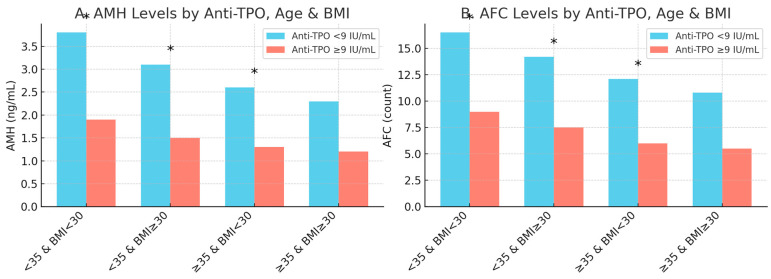
Anti-TPO’s effects on AFC and AMH by age and BMI. The differential effects of anti-TPO positivity (≥9 IU/mL) on the ovarian reserve markers anti-Müllerian hormone (AMH, Panel A) and antral follicle count (AFC, Panel B) across combined age/BMI subgroups. Statistically significant differences (*p* < 0.05) are marked with an asterisk (*).

Between anti-TPO and E2. These findings were consistent across stratified age groups, with stronger negative correlations observed in women < 35 years and in the non-obese subgroup (Figure 2).

Panel A shows that anti-TPO-positive women had significantly lower AMH levels compared to their anti-TPO-negative counterparts in all subgroups except for the oldest and heaviest group (≥35 years old and BMI ≥30). Similarly, Panel B shows that anti-TPO positivity was significantly associated with lower AFC values in all but the ≥35-year-old and BMI ≥ 30 group, where the difference was not significant. These findings suggest that the negative impact of thyroid autoimmunity on ovarian reserve is more pronounced in younger and non-obese women.

*p*-values for AMH and AFC comparisons between anti-TPO groups remained < 0.05 among women aged < 40 years but not in older age groups. Regression analyses indicated that the effects of anti-TPO diminished with increasing age (AMH interaction β = +0.00048, *p* = 0.008; AFC interaction β = +0.00153, *p* = 0.002). Analyses were repeated in BMI-stratified subgroups (<30 vs. ≥30 kg/m^2^). In non-obese women (*n* = 1278), AMH and AFC levels were significantly lower in anti-TPO ≥ 9 patients (AMH β = −0.00297, *p* = 0.001; AFC β = −0.412, *p* = 0.003). In obese women (*n* = 182), there was an association with PCOS (*n* = 342, 23.4%), while levels of anti-TPO were less impactful. Anti-TPO levels did not have a significant effect on AMH (*p* = 0.116) or AFC (*p* = 0.430), probably because this group already has a high ovarian reserve. In women without PCOS (*n* = 1118), anti-TPO was strongly linked to lower AMH (β = −0.0018, *p* < 0.0001) and AFC (β = −0.33, *p* = 0.001), highlighting how thyroid autoimmunity affects those without PCOS. In women who do not have PCOS (*n* = 1118), anti-TPO was strongly connected to lower AMH (β = −0.0018, *p* < 0.0001) and AFC (β = −0.33, *p* = 0.001), demonstrating how thyroid autoimmunity impacts those without PCOS. This study highlights the association between anti-TPO positivity and PCOS. Anti-TPO positivity was significantly less frequent in the PCOS group (22 out of 342, 1.5% of total cases) compared to the non-PCOS group (235 out of 1118, 16.1% of total cases). Conversely, anti-TPO negativity was more prevalent among PCOS patients (320 out of 342, 21.9%) than anti-TPO positivity (1.5%). These findings suggest that anti-TPO positivity is significantly lower in women with PCOS compared to those without.

**Figure 3:**
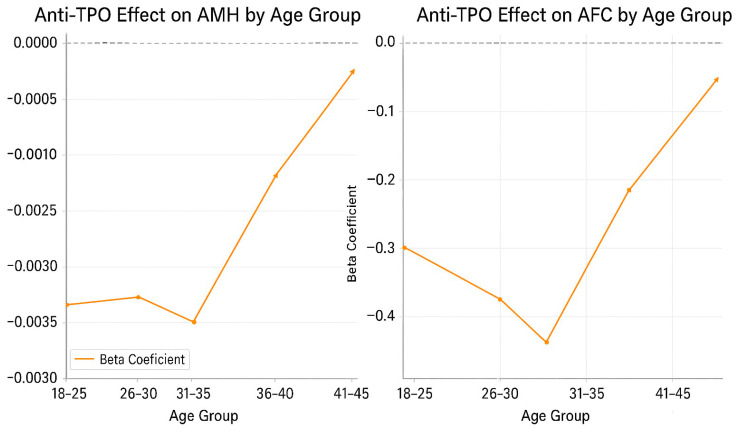
Age-stratified regression coefficients (β) for the effect of anti-TPO on AMH and AFC.

Age-stratified beta coefficients for the association between anti-TPO positivity (≥9 IU/mL) and ovarian reserve markers. The left plot illustrates the effect on anti-Müllerian hormone (AMH), and the right plot shows the effect on antral follicle count (AFC). Both graphs indicate a progressively decreasing negative impact of anti-TPO as age increases, with the most pronounced differences observed in the 30–35-year-old age group (AMH β = −0.0035, *p* = 0.017; AFC β = −0.52, *p* = 0.023). No significant differences were observed in the 40–45-year-old age group, suggesting that physiological decline in ovarian reserve with age may mask the effect of thyroid autoimmunity. These findings highlight the need for age-specific risk stratification when assessing autoimmune-related reproductive compromise (Figure 3).

Two logistic regression models were used to evaluate the predictive value of anti-TPO for diminished ovarian reserve (DOR): AMH < 1 ng/mL: OR = 3.13 [95% CI: 2.03–4.83], *p* < 0.0001; AFC < 5: OR = 6.48 [95% CI: 3.82–11.00], *p* < 0.0001. These associations persisted after adjusting for age, BMI, and TSH. The effect was strongest in non-obese, PCOS-negative women < 35 years. Higher levels of anti-TPO (≥9 IU/mL) were associated with lower AMH and AFC, higher FSH, and a greater chance of having reduced ovarian reserve. These effects were most pronounced in younger (<35), non-obese, and PCOS-negative women. Age moderated the effect of anti-TPO, with a diminished impact in women > 40 years of age. Logistic and linear models provide consistent support for anti-TPO as an independent risk factor for ovarian compromise.

**Figure 4:**
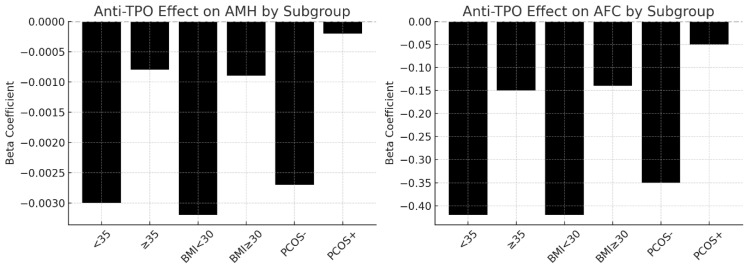
Anti-TPO’s effects on ovarian reserves across subgroups (<35 vs. ≥35).

The beta coefficients from multivariate linear regression analyses showing the effect of anti-TPO positivity on AMH (left panel) and AFC (right panel) across clinical subgroups. Significant negative effects were observed in women under 35, non-obese women (BMI < 30), and women without PCOS. In contrast, the association was weaker and statistically non-significant in women aged ≥ 35 years, those with obesity (BMI ≥ 30), and those with PCOS. These findings suggest that the detrimental impact of thyroid autoimmunity on ovarian reserve is more pronounced in younger, non-obese women without PCOS (Figure 4).

**Figure 5:**
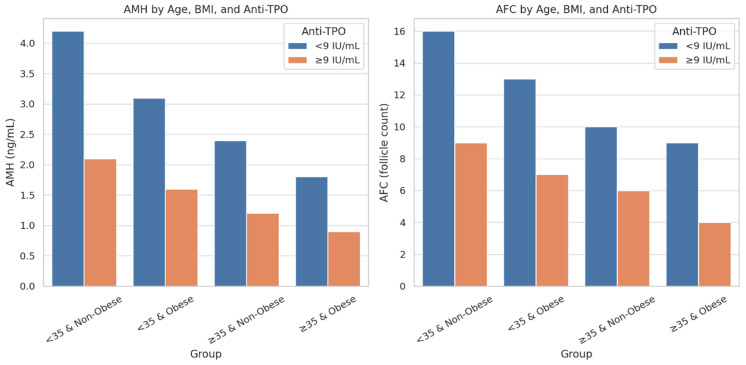
Comparison of AMH and AFC levels by age, BMI, and anti-TPO status.

Differences in anti-Müllerian Hormone (AMH) and antral follicle count (AFC) levels across four stratified categories, <35 and non-obese, <35 and obese, ≥35 and non-obese, and ≥35 and obese, further separated by anti-TPO status (<9 IU/mL vs. ≥9 IU/mL). A marked decline in AMH and AFC is evident in the anti-TPO ≥ 9 IU/mL subgroups, particularly among younger, non-obese individuals, suggesting stronger autoimmune effects in this demographic. These results highlight the age- and BMI-dependent impact of thyroid autoimmunity on ovarian reserve (Figure 5).

**Figure 6:**
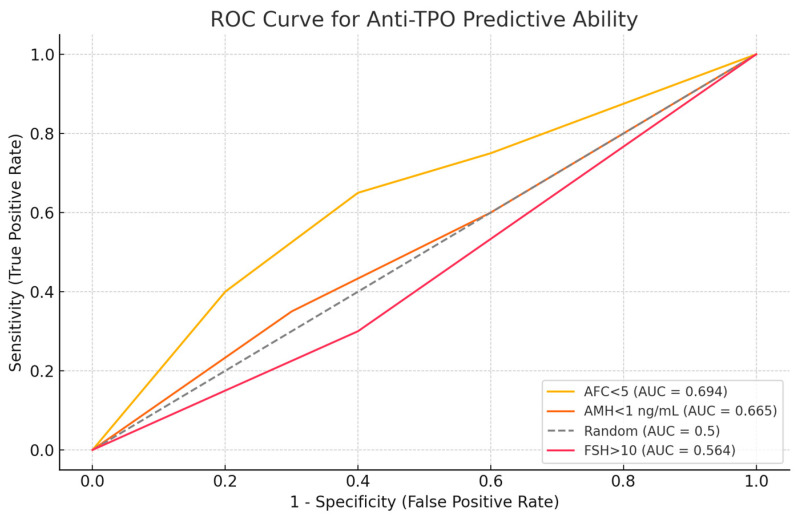
ROC curve analysis of anti-TPO’s predictive ability.

The ROC curve analysis for all patients, including women with PCOS and obesity, indicated that anti-TPO levels were most effective at predicting AFC < 5 (AUC = 0.694) and AMH < 1 ng/mL (AUC = 0.665). However, the ability to predict FSH > 10 was weaker (AUC =0.564). The ability to predict FSH > 10 was not as strong (AUC = 0.564). The predictive ability for FSH > 10 was weaker (AUC = 0.564). Optimal anti-TPO cutoff values were determined to be >10 IU/mL for AFC and >3 IU/mL for both AMH and FSH. ROC analysis yielded AUC 0.68 (95% CI 0.64–0.72; *p* < 0.001) with an optimal cutoff near 9–10 IU/mL (Figure 6).

Receiver operating characteristic (ROC) curve illustrating the discriminatory ability of anti-TPO levels for predicting diminished ovarian reserve indicators. Anti-TPO demonstrated moderate predictive performance for AFC < 5 (AUC = 0.694) and AMH < 1 ng/mL (AUC = 0.665) and lower predictive accuracy for FSH > 10 mIU/mL (AUC = 0.564). 

**Figure 7:**
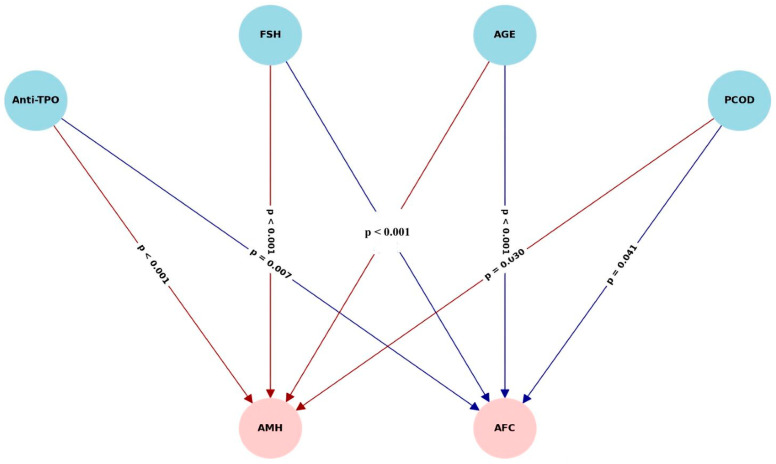
Path diagram depicting structural relationships among anti-TPO, TSH, BMI, age, AMH, and AFC.

Structural equation modeling (SEM) demonstrated that anti-TPO levels exert significant direct negative effects on both AMH (β = −0.015, *p* < 0.001) and AFC (β = −0.39, *p* = 0.002). These associations were independent of TSH levels, suggesting a thyroid-autoimmunity-specific effect rather than one mediated by thyroid hormone alterations. The analysis did not reveal any significant indirect effects through BMI or TSH, reinforcing the direct autoimmune pathway hypothesis. Among all variables in the model, age emerged as the strongest mediator, substantially influencing both AMH and AFC, consistent with the established literature on reproductive aging. These findings underscore the primary immunologic pathway linking anti-TPO antibodies to ovarian reserve depletion, especially when confounders, such as BMI and thyroid function, are accounted for. The multivariate model revealed significant direct effects of anti-TPO, age, and their interactions on FSH levels. While BMI and PCOS alone had marginal effects, higher-order interactions demonstrated that the impact of thyroid autoimmunity on gonadotropin regulation is more pronounced in younger, non-obese, and PCOS-negative individuals. These findings support the hypothesis that FSH elevation observed in anti-TPO-positive women may reflect early follicular depletion independent of TSH modulation (Figure 7).

Results of structural equation modeling evaluating the direct and indirect effects of anti-TPO on anti-Müllerian hormone (AMH) and antral follicle count (AFC). Solid lines represent significant direct effects. Anti-TPO exerted a strong direct negative effect on both AMH (β = −0.015, *p* < 0.001) and AFC (β = −0.39, *p* = 0.002) independent of TSH. Indirect effects through BMI and TSH were non-significant. Age was a dominant covariate affecting both AMH and AFC, reflecting its well-established role in reproductive aging.

## 3. Discussion

This study provides compelling evidence that elevated anti-TPO antibody levels are independently associated with impaired ovarian reserve, even in women with normal thyroid function. Consistent with prior studies, our findings highlight significant reductions in AMH and AFC, along with subtle increases in FSH, among anti-TPO-positive individuals. These observations reinforce the role of thyroid autoimmunity (TAI) as a pathophysiological mechanism in reproductive aging, extending beyond classical hypothyroidism and supporting the growing emphasis on subclinical markers in fertility assessments [1,3].

Anti-TPO positivity, present in 18.4% of our cohort, closely parallels rates reported in other studies examining infertile populations, where estimates range from 18% to 27% [1,3,13]. Huang et al. noted a 20% TAI prevalence in infertile women without overt hypothyroidism, and Domniz and Meirow identified anti-TPO in 24–27% of women with premature ovarian insufficiency (POI), positioning anti-TPO as a prognostic biomarker for autoimmune reproductive compromise [3,13]. Our data demonstrate that these associations persist after controlling for age, BMI, and PCOS, suggesting a direct autoimmune insult on ovarian function.

Our analysis revealed notable interactions between PCOS and TAI. PCOS is characterized by chronic low-grade inflammation, insulin resistance, and anovulation, often accompanied by elevated AMH and AFC due to impaired follicular recruitment [15,16,17]. These hormonal elevations can obscure early autoimmune-related ovarian damage. Our subgroup analysis confirmed that anti-TPO’s effects on AMH and AFC were less pronounced in PCOS patients, particularly in younger individuals. However, with advancing age or coexisting obesity, this masking effect diminished, leading to accelerated ovarian aging [15,16,18]. These results align with findings from Adamska et al. and Huang et al., who reported that while anti-TPO positivity is less frequent in PCOS, its presence correlates with poorer ovarian reserve [19,20]. The bidirectional relationship between PCOS and thyroid autoimmunity is complex and potentially driven by shared genetic, metabolic, and immunologic mechanisms [20,21]. Both PCOS and autoimmune thyroid disease share common pathophysiological pathways including insulin resistance, chronic low-grade inflammation, and possibly shared genetic predispositions such as polymorphisms in the FSH receptor, PPAR-γ, and immune-regulatory genes. These overlapping mechanisms might explain the bidirectional relationship observed between PCOS and thyroid autoimmunity [22,23].

Obesity is as a critical modifier in the autoimmune–reproductive axis [24,25]. Our analysis, consistent with reports by Altuntaş et al. and Croce et al., shows that while obesity elevates AMH and AFC in early reproductive years, this effect diminishes after 30 years of age, resulting in a steeper decline in ovarian reserve among anti-TPO-positive individuals [14,24]. Adipose tissue acts as an endocrine organ, contributing to systemic inflammation and cytokine production, which may synergize with autoimmune pathways. Interestingly, while BMI significantly modulates AMH and AFC levels, our multivariate models indicate that it does not fully mediate anti-TPO’s impact, underscoring a distinct and independent autoimmune effect. These findings support prior conclusions that obesity may attenuate but not eliminate the reproductive risks conferred by thyroid autoimmunity [4,5,26,27]. Adipose tissue is an active endocrine organ that secretes pro-inflammatory cytokines such as TNF-α, IL-6, and leptin, which may exacerbate thyroid autoimmunity and contribute to ovarian dysfunction. Chronic inflammation in obese women could amplify autoimmune responses, accelerating follicular loss and impairing ovarian reserve [28].

Our path analysis further confirmed direct effects of anti-TPO on both AMH and AFC, independent of TSH and BMI. This underscores the notion that anti-thyroid antibodies may exert local cytotoxic effects on ovarian granulosa cells, impairing folliculogenesis and contributing to follicular atresia [4,5,6,12,29,30]. AMH emerged as a more sensitive biomarker than AFC, likely due to its endocrine regulation, while FSH remained largely unaffected, reflecting its regulation at the hypothalamic–pituitary level [4,5,6,12,26,27,28]. These mechanistic insights align with accumulating data showing that thyroid autoimmunity may induce granulosa cell apoptosis, immune cell infiltration, oxidative stress, and ovarian microvascular injury, ultimately compromising ovarian reserve [17,21,31,32,33,34].

Anti-TPO also appears to serve as a subclinical indicator of broader metabolic disruption. Previous studies have reported that anti-TPO positivity precedes insulin resistance by approximately one year, highlighting its potential utility as an early warning marker for metabolic and reproductive compromise [26,32,35]. Given the established link between insulin resistance and ovarian dysfunction, especially in PCOS, anti-TPO screening may offer an upstream approach to risk stratification. These results support a paradigm shift in infertility workups and the integration of thyroid antibody profiling with hormonal and metabolic assessments, particularly in women under 35 years of age with unexplained infertility or preserved TSH levels.

Regional data add another layer of clinical relevance. Elevated anti-TPO prevalence in PCOS populations across specific regions, including our study region, may reflect environmental iodine intake, genetic susceptibility, or laboratory variations in assay sensitivity. Regardless, these findings support the broader application of thyroid antibody testing in reproductive endocrinology beyond patients with overt hypothyroidism [36].

Our study also evaluated ovarian reserve trajectories across age and BMI strata. The steepest declines in AMH and AFC occurred in anti-TPO-positive women over the age of 30, with or without PCOS. This age-dependent vulnerability, coupled with autoimmune factors, could represent an early phase of ovarian senescence [15,21,36]. The synergy between age-related immune changes and autoimmune activation may amplify follicular loss, especially in genetically predisposed individuals [6,29,32,37]. Anti-TPO’s predictive power for DOR, defined as AMH < 1 ng/mL or AFC < 5, remained robust in logistic models, even after adjusting for TSH. ROC analyses supported these findings, identifying anti-TPO as a moderately effective predictor of DOR, with optimal cutoff values around 9–10 IU/mL.

Anti-TPO antibodies may contribute to impaired ovarian function through immune-mediated mechanisms such as cross-reactivity between thyroid and ovarian antigens, complement activation, and local inflammation leading to follicular atresia. Studies have shown the presence of thyroid antibodies in ovarian tissue, suggesting possible autoimmune-mediated follicular depletion [38].

Although the mean age difference between the groups was not statistically significant, even minor age variations may influence ovarian reserve parameters such as AMH and AFC, given the age-dependent decline in follicular quantity and quality

Our clinical findings indicate that anti-TPO should be regarded as a significant marker of ovarian dysfunction, rather than a benign spectator. In younger, euthyroid women with fertility concerns, anti-TPO testing may facilitate earlier diagnosis, inform treatment timelines, and prompt fertility preservation counseling. Integrating anti-TPO into fertility risk models could improve individualized care and help identify women who may benefit from immunological or endocrinological interventions

## 4. Clinical Recommendations

The study recommends routine anti-TPO antibody screening for infertile women under 35, even with normal thyroid hormone levels. This integrated approach stratifies infertility risk, personalizes fertility care, and could benefit young, non-obese women.

## 5. Study Strengths and Limitations

The study on thyroid autoimmunity and ovarian function, despite limitations like retrospective nature and single-center setting, offers valuable insights into the relationship between thyroid autoimmunity and ovarian function, requiring further prospective, multicenter investigations.

## 6. Future Directions

The study recommends further research on thyroid autoimmunity in female reproductive health, including cohort studies, genomic profiling, and incorporating autoimmune markers into fertility evaluations for improved treatment planning.

## 7. Conclusions

Anti-TPO antibodies are moderately but independently associated with diminished ovarian reserve, irrespective of thyroid hormone status. Effects are most evident in younger, non-obese, PCOS-negative women but may remain concealed in PCOS or obesity until later reproductive years. Anti-TPO testing can enhance infertility risk stratification as part of a multifactorial evaluation but should not be used in isolation for diagnostic purposes.

## Figures and Tables

**Table 1:** Baseline Demographic and Laboratory Characteristics by Anti-TPO Status.

	Anti-TPO (−) (*n* = 1203)	Anti-TPO (+) (*n* = 257)	*p* Value
**Age (year)**	31.45 ± 5.66	32.61 ± 5.76	0.033
**BMI (Kg/m^2^**)	26.15 ± 3.48	26.37 ± 3.69	0.469
**Obesity BMI (Kg/m^2^**) **≥ 30 (*n*)**	144(17.7%)	38(14.8%)	0.213
**Duration of infertility (mounts)**	61.19 ± 18.84	61.24 ± 19.17	0.963
**Number of antral follicle count (AFC) (*n*)**	15.88 ± 8.18	8.18 ± 5.06	<0.001 *
**Anti-Mullerian hormone (AMH) ng/Ml**	3.34 ± 3.04	1.47 ± 1.53	<0.001 *
**Day 3 Follicle-stimulating hormone (FSH), mIU/ml**	8.06 ± 4.79	9.40 ± 6.21	0.001 *
**Day 3 Luteinizing hormone (LH), mIU/ml**	6.65 ± 4.42	5.92 ± 3.85	0.015 *
**Day 3 Estradiol (E2), pg/ml**	40.51 ± 26.36	39.85 ± 22.60	0.741
**Troide-stimulating hormone (TSH), mIU/ml**	1.93 ± 0.83	2.22 ± 1.11	0.002 *
**Free Triiyodotironin (Ft3), pg/mL**	3.48 ± 0.41	3.46 ± 0.42	0.482
**Free Tiroksin (Ft4), ng/dL**	0.93 ± 0.13	0.92 ± 0.14	0.176

Data are presented as the mean ± SD for normally distributed variables (BMI, FT3, FT4) and median (IQR) for non-normally distributed variables. Comparisons between groups were performed using Student’s *t*-test or Mann–Whitney U test for continuous variables and χ^2^ test for categorical variables. * *p* < 0.05 considered statistically significant.

## Data Availability

The data used for this manuscript are available upon reasonable request of the corresponding author: Sefa Arlıer (Email: sefaarlier@gmail.com).

## Abbreviations

AMH	Anti-Müllerian Hormone
AFC	Antral Follicle Count
BMI	Body mass index
DOR	Diminished ovarian reserve
E2	Estradiol
Ft3	Free Triiyodotironin
Ft4	Free Tiroksin
HT	Hashimoto’s thyroiditis
hCG	Human chorionic gonadotropin
IVF	İn vitro fertilization
PCOS	Polycystic ovary syndrome
POI	Premature ovarian insufficiency
FSH	Follicle-Stimulating Hormone
LH	Luteinizing hormone
TAI	Thyroid autoimmunity
Anti-Tpo	Thyroid peroxidase antibodies
TVS	Transvaginal ultrasound
